# MicrocrackAttentionNext: Advancing Microcrack Detection in Wave Field Analysis Using Deep Neural Networks Through Feature Visualization

**DOI:** 10.3390/s25072107

**Published:** 2025-03-27

**Authors:** Fatahlla Moreh, Yusuf Hasan, Bilal Zahid Hussain, Mohammad Ammar, Frank Wuttke, Sven Tomforde

**Affiliations:** 1 Department of Geo-Science, Christian Albrechts University, 24118 Kiel, Germany; frank.wuttke@ifg.uni-kiel.de; 2 Department of Computer Engineering, Aligarh Muslim University, Aligarh 202001, India; yusufhasan@zhcet.ac.in (Y.H.); mohammadammar@zhcet.ac.in (M.A.); 3 Department of Electrical and Computer Engineering, Texas A&M University, College Station, TX 77840, USA; zahidhussain909@tamu.edu; 4 Institute of Computer Science, Christian Albrechts University, 24118 Kiel, Germany; st@informatik.uni-kiel.de

**Keywords:** microcrack detection, CNN, spatio–temporal data, feature space visualization, acoustic emission, segmentation, attention

## Abstract

Microcrack detection using deep neural networks (DNNs) through an automated pipeline using wave fields interacting with the damaged areas is highly sought after. However, these high-dimensional spatio–temporal crack data are limited. Moreover, these datasets have large dimensions in the temporal domain. The dataset presents a substantial class imbalance, with crack pixels constituting an average of only 5% of the total pixels per sample. This extreme class imbalance poses a challenge for deep learning models with different microscale cracks, as the network can be biased toward predicting the majority class, generally leading to poor detection accuracy for the under-represented class. This study proposes an asymmetric encoder–decoder network with an adaptive feature reuse block for microcrack detection. The impact of various activation and loss functions are examined through feature space visualisation using the manifold discovery and analysis (MDA) algorithm. The optimized architecture and training methodology achieved an accuracy of 87.74%.

## 1. Introduction

Microcrack detection in materials is of significant importance due to the potential for catastrophic failures, which can lead to substantial financial losses and safety hazards in industries [1,2]. In a number of areas, including materials science, aerospace, and infrastructure, where the existence of small fissures might jeopardize the structural integrity of materials, non-destructive techniques (NDTs) for microcrack detection is imperative [3,4,5]. Conventional techniques for detecting microcracks are frequently labor-intensive and not very scalable [6,7]. Some NDTs, such as visual inspection and dye-penetrant testing, are fairly simple but are limited to surface-level cracks and require intensive labor. Others, such as magnetic particle testing and eddy current testing, are exclusive for some materials. More advanced techniques such as X-ray computed tomography, although quite effective, are relatively expensive and limited to smaller components [8]. Moreover, detecting cracks in complex structures, such as aircraft bodies or intricate machinery components, poses a substantial challenge using conventional methods. The use of acoustic wave-based approaches for crack detection offers a powerful solution, as these methods allow for the analysis of structures that are not easily accessible or too complex to inspect manually. However, acoustic emission-based approaches are prone to background noises, which makes their analysis untractable through data analysis techniques. Deep learning techniques provide a solution to this problem by analyzing huge amounts of data to discern meaningful patterns. Convolutional neural networks (CNNs) are especially good at processing spatial data due to their ability to capture local spatial correlations within an image [9,10]. Segmentation through CNNs is highly sought after as these techniques help localize the objects of interest in the image. These techniques have been extensively used in medical image segmentation and showed a high degree of reliability and accuracy [11].

Nevertheless, standard segmentation methods demonstrate limited performance on this particular dataset, due to the complex spatio–temporal nature of the crack patterns. This becomes even more significant when the cracks represent a minority class in the dataset, leading to poor detection accuracy. The dataset not only presents a severe class imbalance but also introduces additional complexities, including multiple crack sizes, varying crack shapes, and the presence of cracks in different locations. These variations significantly impact the wave propagation behavior, making it challenging for traditional segmentation models to generalize effectively. The model must learn to differentiate between subtle variations in wave interactions caused by different types of cracks while overcoming the bias introduced by dominant non-crack regions. This issue is enhanced when dealing with very small cracks, as they not only lead to data imbalance but may also cause minimal disruption in underlying wave behavior. In such cases, the waves may exhibit minimal changes, making it difficult for the model to detect the cracks accurately. Moreover well-known segmentation models such as U-Net are not compatible with these datasets, as these have both temporal and spatial dimensions. The model not only has to detect patterns across spatial dimensions but also across temporal domains, and the U-Net model is used for the image to image translation problem where the input and output dimensions are equal.

This challenge necessitates the development of a more tailored custom model. The proposed MicrocrackAttentionNext is designed to overcome the limitations of vanilla models such as U-Net by incorporating enhanced spatial and temporal feature extraction. Unlike U-Net [12] where the input and target share the same modality (image-to-image translation). The proposed model processes spatio–temporal input data and outputs spatial crack predictions, enabling it to handle more complex data while improving micro-scale detection accuracy. The asymmetric encoder–decoder structure with attention layers is particularly effective, as it focuses on capturing critical crack patterns rather than relying heavily on skip connections. The attention mechanism ensures that the model prioritizes the time steps when the waves interact with the cracks, improving detection precision. To further evaluate the effect of activation functions and different losses, the proposed work employs feature map analysis. The extent of the influence of different activations is difficult to determine against conventional metrics such as accuracy and F1 score. Hence, it is imperative to analyze the internal dynamics of the model.

Methods such as principal component analysis, t-SNE [13], and UMAP [14] are used to analyze the higher dimensional feature maps of these black box models against the target. However, these methods provide little to no insight when used on segmentation problems. This study uses the recently proposed manifold discovery analysis (MDA) [15] to qualitatively assess the impacts of various activation functions. Moreover, through this, the proposed study is able to analyze the effect activations had on the feature maps of the model, allowing us to choose the best activation function for the given problem. These activation functions aim to strike a delicate balance between adaptability and computational efficiency, essential considerations in the micro-material domain, where capturing fine details is crucial for accurate crack detection. Empirical exploration and meticulous fine-tuning of these activation functions is imperative to identify the optimal choice that aligns with the distinctive characteristics of micro-material images. Ultimately, an effective approach to crack detection in micro-materials relies on the thoughtful selection and optimization of activation functions within the CNN architecture.

The primary contributions of this paper are the following:

- Introducing MicrocrackAttentionNext—an improvement over [16]—and introduction of Adaptive Feature Utilization Block for efficient feature utilization.
- Analysis of the impact of activation functions on the performance of MicrocrackAttentionNext through Manifold Discovery and Analysis in the context of microcrack detection.

The paper’s structure is outlined in the following manner: Section 2 provides a concise overview of relevant studies. Section 3 deals with the dataset used and the proposed methodology. Section 4 explains the experiments conducted. The assessment of the performance of the proposed system and the results obtained are included in Section 5. The concluding remarks and future works are presented in Section 6.

## 2. Related Works

This section primarily examines non-invasive methods for the detection of microcracks in numerical data. The first two paragraphs present methodologies rooted in classical foundations, followed by an exploration of more recent deep-learning approaches.

In their study, Punnose et al. [17] conducted an experimental evaluation of the time-of-flight diffraction (TOFD) technique for accurately sizing surface-breaking cracks. Using steel test blocks with vertical and inclined slits of varying heights, the researchers employed TOFD equipment with 45° longitudinal angle beam probes to scan and analyze these simulated defects. The findings demonstrated that TOFD could measure crack depths with an average error of ±0.13 mm for vertical slits and ±0.05 mm for inclined slits, and crack lengths with an average error of ±0.36 mm and ±0.29 mm, respectively. However, the study also identified challenges in detecting defects less than 2 mm deep due to lateral wave interference and limited time resolution near the surface. The findings demonstrate TOFD’s capability for accurate crack measurement and point to the necessity of enhancing its sensitivity to shallow flaws.

Kou et al. [18] proposed a fully noncontact nonlinear ultrasonic testing (NUT) method using laser ultrasonics for detecting closed surface cracks. Traditional ultrasonic techniques struggle with closed cracks due to minimal wave scattering. This study employs a pulsed laser grating source to generate narrowband surface acoustic waves (SAWs) and uses a nonlinear numerical model based on the finite element method (FEM) to simulate higher harmonic generation.

Conventional techniques for detecting microcracks are frequently labour-intensive and not very scalable. Deep learning and convolutional neural networks (CNNs), in particular, have become a potent and effective technique for microcrack detection automation in recent years [19,20,21].

Tran et al. [22] applied 1D convolutional neural networks (1D CNNs) for structural damage detection, utilizing acceleration signals to detect cracks in numerical steel beam models. Their approach showed high detection accuracy, comparable to more complex methods, by processing time-series data and extracting key features related to structural changes. While they focused on single-dimensional data, the proposed research extends this by using multi-dimensional spatio-temporal data, which includes wave propagation across the material. This allows for more detailed analysis, capturing both spatial and temporal interactions crucial for detecting microcracks.

Jiang et al. [23] combined 1D CNNs with support vector machines (SVMs) to enhance structural damage detection. The 1D CNNs were used to localize damage, while SVMs focused on classifying the severity, benefiting from the strengths of both models in feature extraction and small-sample learning.

Barbosh et al. [24] used acoustic emission (AE) waveforms and DenseNet to detect and localize the crack. The localization was conducted to determine whether the crack was close to the sensor or far away. The cracks were also classified into severe and less severe cracks.

Moreover, Li et al. [25] proposed a GM-ResNet-based approach to enhance crack detection, utilizing ResNet-34 as the foundational network. To address challenges in global and local information assimilation, a global attention mechanism was incorporated for optimized feature extraction. Recognizing limitations in ResNet-34, the fully connected layer was replaced with a multilayer fully connected neural network (MFCNN), featuring multiple layers, including batch normalization and Leaky ReLU nonlinearity. This innovative substitution significantly improved the model’s ability to capture complex data distributions and patterns, enhancing feature extraction and representation capabilities while preventing overfitting during training.

Moreh et al. [16] explore the use of DNN for automated crack detection in structures using seismic wave signals. The authors improve on previous asymmetric encoder–decoder models by experimenting with different encoder backbones and decoder layers. The best combination was found to be the 1D-DenseNet encoder and the Transpose Convolutions as decoders. The proposed model achieved an accuracy of 83.68% with a total parameter count of 1.393 million.

This study builds upon the foundational contributions of Moreh et al. [16,26,27], extending their methodologies to a broader scope. The existing body of work in this field remains relatively sparse, with few studies addressing crack segmentation through the specific approach employed in this research.

## 3. Method

The proposed work targets the detection of various microcrack sizes and locations within seismic wave field numerical data. For this purpose, MicrocrackAttentionNext extracts crucial signals from the data to identify and detect those cracks. This is done by learning the temporal representations, followed by spatial representations. These encoded data are then passed through the decoder to achieve semantic spatial segmentation.

The following section describes the seismic wave data followed by the architecture of the proposed MicrocrackAttentionNext model and, subsequently, the training procedure used.

### 3.1. Wave Field Data

**Figure 1:**
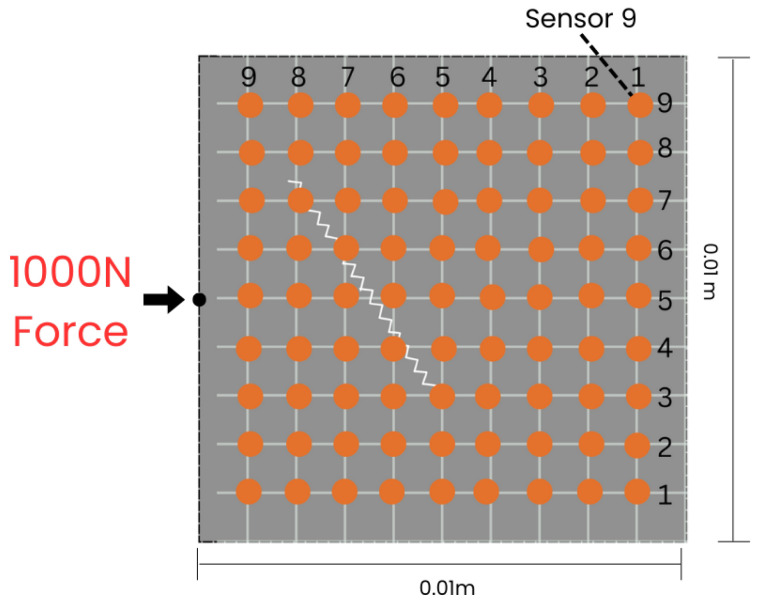
Arrangement of the 9 × 9 sensor array in the sample domain.

**Figure 2:**
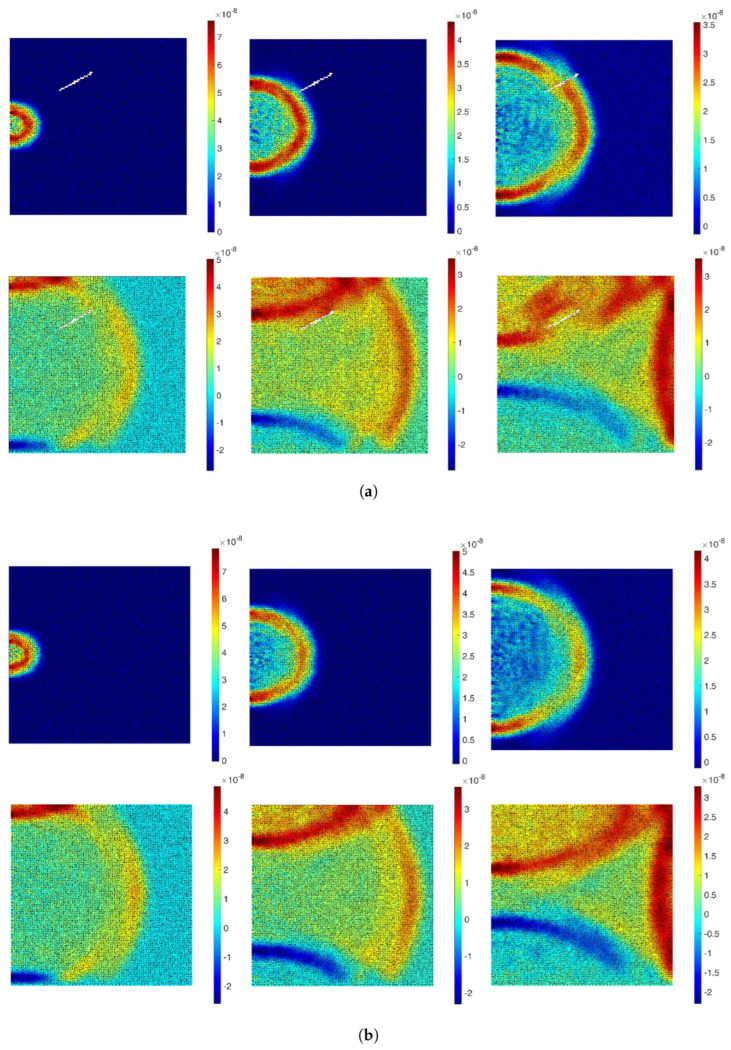
The 6 frames (100 time-steps interval, from left to right) of a displacement wave propagation inside the defined plate with [28] (**a**) crack and (**b**) no crack.

**Figure 3:**
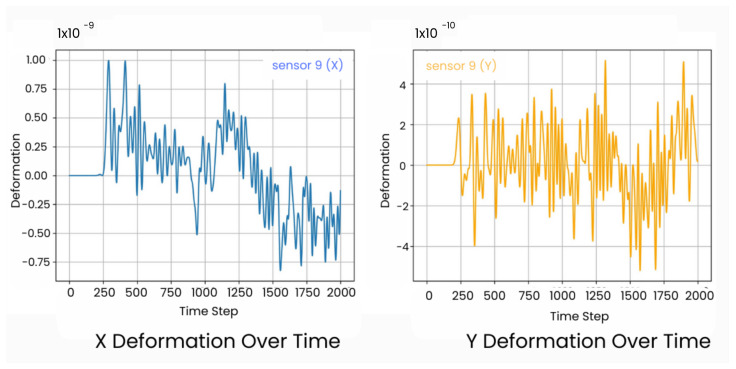
The 2D—visualization of time signal received by sensor 9 in a sample.

The wave field dataset utilized in this work, while effective for crack detection, presents some limitations in terms of data dimensionality. These datasets are characterized by large temporal dimensions, which increases the complexity of data processing and model training. The dataset consists of wave field numerical data for homogeneous 2D plates, where each plate is modeled with lattice particles that share consistent properties, such as density and Young’s Modulus. The modeling of structural systems is achieved using Voronoi–Delaunay meshing algorithms within the lattice element method (LEM). Lattice nodes, representing unit cell centers, are connected by beams capable of handling normal forces (*N*), shear forces (*V*), and bending moments (*M*). If the strain energy $U_{e}$ in an element exceeds a predefined threshold $U_{\mathrm{th}}$, the element undergoes stiffness reduction or removal, simulating failure states. To simulate wave propagation through the material, an external force of 1000 N is applied at the midpoint of the left boundary (Figure 1), ensuring that the waves propagate across the entire plate, interacting with both non-crack (Figure 2b) and crack regions (Figure 2a), the wave deflects when it interacts with the crack, and the displacement is recorded by the sensors. The resulting displacements in both the x- and y-directions are recorded over 2000 time steps, capturing detailed temporal changes in the wave field. Figure 3 shows 2D visualization of the time signal received by sensor 9 in a sample. These displacements are measured by a 9 × 9 (81) sensor grid (Figure 1) uniformly distributed across the material, resulting in a wave field dataset with dimensions of 2 × 81 × 2000. This approach provides spatio-temporal data that captures the interaction between the propagating waves and the cracks, allowing for in-depth analysis of crack detection model performance. This approach provides spatio-temporal data that capture the interaction between the propagating waves and the cracks, allowing for in-depth analysis of crack detection model performance. The dataset used for training and evaluation consists of a total of 7480 samples, with 4810 allocated for training, 910 for validation, and 1760 for testing.

A major challenge arises from the severe class imbalance present in the dataset. On average, only 5% of the total pixels represent cracks, with the remaining majority belonging to intact, non-crack regions. This imbalance poses a substantial obstacle for deep learning models, which are prone to bias toward the majority class. As a result, the models tend to predict non-crack regions more frequently, leading to suboptimal detection accuracy for the minority class (crack regions). Addressing this issue requires careful design of the model and training process to ensure that the network can effectively learn from the minority class, and accurately identify crack regions without being overpowered by the majority class imbalance. Moreover, the effect of different activations and loss functions are assessed both quantitatively and qualitatively.

### 3.2. MicrocrackAttentionNext Model Architecture

**Figure 4:**
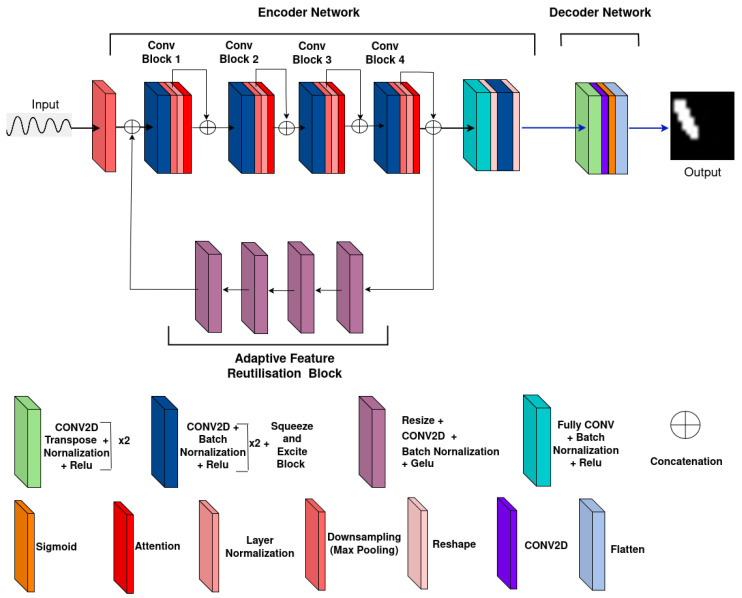
MicrocrackAttentionNext model architecture.

MicrocrackAttentionNext, shown in Figure 4, is an asymmetric encoder–decoder network. The input to the model is a tensor with shape $X\in R^{C_{\mathrm{in}}\times T\times S}$, where $C_{\mathrm{in}}=2$ represents the input channels corresponding to the *x* and *y* components of wave data, T=2000 is the temporal dimension, and S=81 corresponds to the spatial dimension, which is a flattened 9×9 sensor grid. To reduce computational complexity and focus on salient temporal features, the network uses an initial max pooling layer with a kernel size of (4,1). This layer transforms the input tensor X to $X_{1}\in R^{2\times 500\times 81}$ by downsampling the temporal dimension from 2000 to $T_{1}=500$. This reduction is crucial as it reduces the amount of data the subsequent layers need to process.

The encoder is composed of four convolutional blocks [26,27], each designed to progressively extract higher-level features from the input data. The first convolutional block applies two 1D convolutional layers with kernel sizes (3,1) and padding (1,0), which maintain the spatial dimensions while expanding the channel dimension from 2 to 16. These layers are followed by batch normalization and activation functions, introducing non-linearity. A squeeze-and-excitation (SE) module is then applied, which recalibrates channel-wise feature responses by modeling interdependencies between channels. This module enhances the representational power of the network by allowing it to focus on the most informative features. Following the first 1D convolutional block, a max pooling layer with a kernel size of (2,1) further reduces the temporal dimension from 500 to $T_{2}=250$. Group normalization is applied to the data, normalizing across channels and improving convergence during training. An AttentionLayer computes self-attention over the temporal and spatial dimensions, enabling the network to weigh different parts of the input differently. This attention mechanism is essential for focusing on relevant features and capturing dependencies across the data. A residual connection adds the attention output back to the original input, facilitating better gradient flow and mitigating issues such as vanishing gradients [29,30].

This pattern repeats in the subsequent convolutional blocks, with each block increasing the number of channels (from 16 to 32, 32 to 64, and 64 to 128) and further reducing the temporal dimension (from 250 to 125, 125 to 62, and 62 to 31) through additional pooling layers. The consistent use of (3,1) kernels ensures effective temporal feature extraction while preserving spatial dimensions. SE modules and attention mechanisms are integrated throughout. Feature maps are upsampled and reintroduced to the Conv1 block through a self-attention module (SAM)-inspired mechanism [26], enabling the decoder backbone to reuse features and increase model performance. The feedback mechanism employs bilinear interpolation for resizing and utilizes Conv2D layers to selectively regulate the features propagated back into the network giving it the alias of Adaptive Feature Reutilization block.

At the bottleneck of the network, a convolutional layer with a large kernel size of (31,1) is employed, covering the entire temporal dimension $T_{5}=31$. This layer transforms the tensor to $X_{\mathrm{bottleneck}}\in R^{B\times 128\times 1\times 81}$, capturing long-range temporal dependencies and encapsulating high-level temporal information into a compact form. Batch normalization and activation are applied to maintain training stability and introduce non-linearity.

The decoder begins by reshaping this bottleneck tensor into a spatial grid $X_{\mathrm{reshaped}}\in R^{128\times 9\times 9}$, reorganizing the data for spatial processing. A point-wise convolution reduces the channel dimension from 128 to 16, preparing the data for upsampling. The network then uses transposed convolutional layers to reconstruct the spatial dimensions progressively. The first transposed convolution upsamples the spatial dimensions from 9×9 to 18×18 and reduces the channel dimension from 16 to 8. The second transposed convolution further upsamples the dimensions to 16×16, maintaining the channel count at 8. Each transposed convolution is followed by batch normalization to ensure stable learning and effective non-linear transformations.

Finally, a point-wise convolution reduces the channel dimension from 8 to 1, and a sigmoid activation function scales the output to the range [0,1]. The output tensor $Y\in R^{1\times 16\times 16}$ represents the reconstructed spatial data, which are then flattened into a vector $Y_{\mathrm{flat}}\in R^{256}$ (since 16×16=256), making it suitable for downstream tasks. Figure 4 shows the proposed model architecture.

The architectural choices in MicrocrackAttentionNext are designed to balance feature extraction capability and computational efficiency. The initial temporal downsampling reduces the data size, allowing the network to process longer sequences without excessive computational overhead. The 1D convolutional blocks with increasing channel dimensions enable the extraction of hierarchical features in the temporal domain without mixing the spatial component. It is found that learning temporal and spatial components separately enables the model to learn better representations while being computationally efficient. The squeeze-and-excitation layers optimize the network’s focus on informative channels, improving feature quality [31,32,33]. Using a large kernel size in the bottleneck layer is an intentional choice to capture long-range temporal dependencies, which are important in sequences where connected events are separated by large time steps [34,35]. The reshaping and upsampling in the decoder reconstruct the spatial dimensions effectively, ensuring that the high-level features extracted by the encoder are used to generate outputs. These architectural choices are arrived at through rigorous experimentation. The following section dives into these experiments in detail.

## 4. Experiments

### 4.1. Architectural Choices

**Figure 5:**
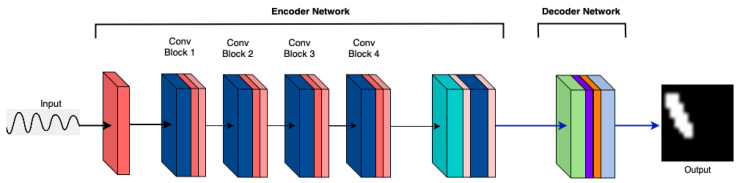
M1: Vanilla (Baseline) model architecture.

The baseline model architecture, MicrocrackAttentionNext, as illustrated in Figure 5, serves as the foundation for evaluating the impact of architectural adjustments. Each modification’s effect on accuracy across crack sizes is analyzed through corresponding accuracy vs. crack size graphs. *M_x_* refers to the specific model variant, where *x* represents the variant index.

**M1: Vanilla (Baseline)** The performance of the vanilla model [27] is depicted in Figure 5, which plots accuracy against architectural modifications for different crack sizes. This has a simple convolution-based encoder. It serves as the baseline for further investigation of the impact of various architectural changes. The vanilla encoder shows satisfactory performance across various crack sizes.

**M2: Adding Adaptive Feature Reuse Block:** The adaptive feature reuse block shows marginal improvement in accuracies across crack sizes. This is due to the increased discriminative nature of the block because of the sigmoid layer in the block’s last layer.

#### 4.1.1. Attention Mechanism Placement

**Figure 6:**
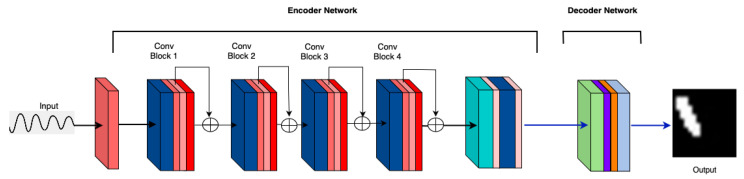
M3: Self-attention layer after convolution block.

**M3: Self Attention Layers:** It is observed that self-attention after convolution blocks (Figure 6) does not affect the accuracies of the model. This is after the skip connections.

**Figure 7:**
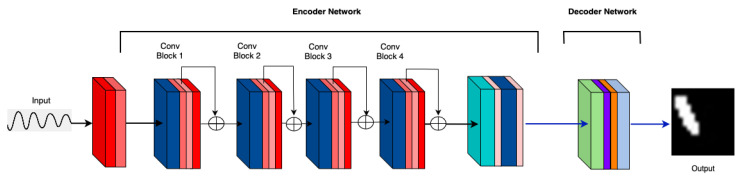
M4: Attention before max pooling in the first layer.

**M4: Attention before Max Pooling in the First Layer: **Figure 7 illustrates the accuracy trends for attention applied before pooling. At Epoch 50, the performance remains suboptimal across different crack sizes. This is probably due to the information bottleneck caused by the attention layers.

**M5: Attention before Max Pooling (Prolonged training):** Prolonged training to Epoch 100 results in even poorer performance across all crack sizes, suggesting that early attention causes severe information bottlenecks for the subsequent layers.

**Figure 8:**
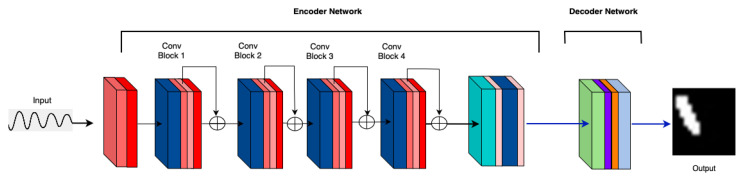
M6: Attention after max pooling in the first layer.

**M6: Attention after Max Pooling:** Performance remains suboptimal for attention after max pooling configuration. Figure 8 shows the performance of the attention after max pooling. This is also caused by the bottleneck issue.

#### 4.1.2. Pooling Variants

**M7: 4x1 Max Pooling and Average Pooling Hybrid:** Using max pooling for the first layer and average pooling in the subsequent layers, the graph indicates virtually no change in the accuracy. This implies that the effects of average pooling is not substantially different from max pooling in this case.

**M8: All Average Pooling:** The results for this configuration reveal slightly higher accuracy for crack sizes >1μm and >2 μm. Otherwise, the graph indicates virtually no change in the accuracy.

**Figure 9:**
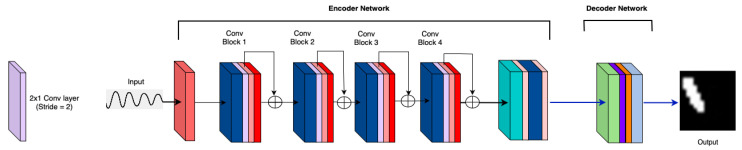
M9: Convolutional pooling layers.

**M9: Convolutional Pooling Layers: **Figure 9 compares the performance of convolutional pooling layers at Epoch 50. The results demonstrate decreased accuracy across all crack sizes, with more dips for smaller cracks. This highlights the advantage of max pooling over convolutional downsampling spatially, as it ensures the preservation of dominant features within each pooling region without introducing additional learnable parameters. By focusing on the maximum value, max pooling effectively captures critical localized details, such as the subtle variations indicative of smaller cracks, which are often diluted by the averaging effect of convolutional downsampling.

**M10: Convolutional Pooling Layers (Prolonged Training):** Prolonged training to Epoch 100 enhances performance slightly, emphasizing the trainable pooling’s ability to adaptively refine features. Overall, the deviation remains within a maximum of 2% for all crack sizes, indicating no major improvements to the model.

#### 4.1.3. Consecutive Attention Layers in the Encoder

**Figure 10:**
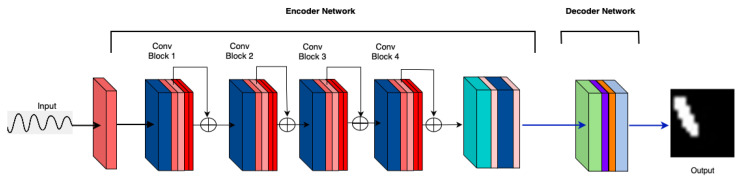
M11: Two attention layers.

**M11: Two Attention Layers (Epoch 50):** The graph in Figure 10 reveals improved accuracy for smaller crack sizes due to the enhanced focus provided by stacked attention layers. The benefits are more pronounced at finer resolutions.

**M12: Two Attention Layers (Prolonged Training):** Prolonged training of this configuration results in improved accuracies; however, it still remains the same across the board.

**Figure 11:**
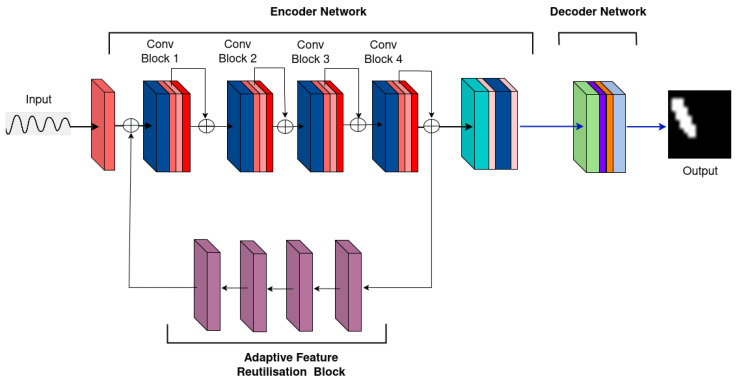
M13: MicrocrackAttentionNext with single FeatureReuse model architecture.

**M13: MicrocrackAttentionNext with single FeatureReuse:** The model shown in Figure 11 demonstrates strong performance across all thresholds. It achieves an overall accuracy of 85.53% for all cracks (≥0) and progressively higher accuracies for larger cracks, reaching 98.29% for cracks >4μm. The single feature reuse mechanism effectively balances the retention of critical features, aiding in accurate detection of both small and large cracks.

**M14: MicrocrackAttentionNext with dual FeatureReuse:** Incorporating feature reuse twice results in slightly lower accuracy compared to the single feature reuse model. The accuracy for all cracks (≥0) drops to 83.24%, with corresponding reductions observed across all thresholds. For cracks > 4 μm, the model achieves 97.2%, showing its capability for detecting larger cracks but indicating potential redundancy or noise in the reused features, which may hinder performance on smaller cracks.

**M15: MicrocrackAttentionNext with dual FeatureReuse and Extended Training:** Extending the training to 100 epochs with two feature reuse steps does not significantly improve performance. The overall accuracy further drops to 82.27%, and the accuracy for larger cracks is >4 μm is 96.3%. The results suggest that prolonged training does not compensate for the challenges introduced by additional feature reuse, particularly in handling smaller cracks effectively.

**Figure 12:**
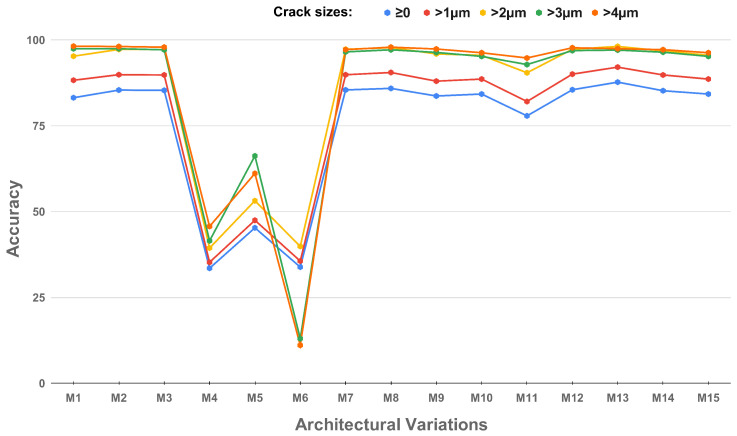
Performance comparison of architectural configurations for crack detection: **M1**: Baseline (MicrocrackAttentionNext 50E), **M2**: Adding Adaptive Feature Reuse Block, **M3**: Self-Attention Layers, **M4**: Attention before Max Pooling in the First Layer, **M5**: Attention before Max Pooling (Prolonged training), **M6**: Attention after Max Pooling, **M7**: 4x1 Max Pooling and Average Pooling Hybrid, **M8**: All Average Pooling, **M9**: Convolutional Pooling Layers, **M10**: Convolutional Pooling Layers (Prolonged training), **M11**: Two Attention Layers (Epoch 50), **M12**: Two Attention Layers (Prolonged Training), **M13**: MicrocrackAttentionNext with single FeatureReuse, **M14**: MicrocrackAttentionNext with dual FeatureReuse, **M15**: MicrocrackAttentionNext with dual FeatureReuse and Extended Training.

Figure 12 shows detection outcomes from the various network variants developed. The performance measures are provided at different threshold levels, spanning from relaxed to stringent detection criteria. At the strictest threshold (>4 μm), the performance values cover a wide range, from a low of 11.09 in configuration M6 to a top value of 98.2 in configuration M1. We select the configuration with the highest metric, M1, as it delivers the best detection performance.

### 4.2. Training Procedure

The model was trained using the Adam optimizer [36] with a learning rate of 0.001 for a total of 50 epochs. Multiple experiments were run on different activation functions and loss functions. The experiments involved evaluating four different activation functions against four loss metrics, resulting in a total of 16 experiments. The activation functions and loss metrics are outlined below. All experiments were conducted on NVIDIA RTX 4090 GPU accelerated systems having Intel Xeon CPU with Tensorflow 2.10 and CUDA 12.0.

#### 4.2.1. Activation Functions

Activation functions are used to introduce non-linearity within neural networks, each offering different advantages for a DL model. The rectified linear unit (ReLU) [37] is defined as ReLU(x)=max(0,x), outputting the input if positive and zero otherwise, thus avoiding vanishing gradient issues. The scaled exponential linear unit (SELU) [38] normalizes outputs automatically, scaling negative inputs with an exponential function and multiplying positive inputs by a fixed constant, where λ=1.0507 and α=1.67326. The Gaussian error linear unit (GELU) [39] employs the Gaussian cumulative distribution function, $\Phi \left(x\right)=\frac{1}{2}\left[1+\mathrm{erf}\left(\frac{x}{\sqrt{2}}\right)\right]$, to probabilistically weigh input significance. GELU smoothly blends linear and non-linear behavior, making it more flexible in capturing complex patterns. The exponential linear unit (ELU) [40] applies ELU(x)=x for positive inputs and $\alpha (e^{x}-1)$ for negatives, mitigating vanishing gradients more effectively than ReLU, and accelerating convergence, with α typically set to 1. Each function enhances network performance through tailored non-linear transformations.

#### 4.2.2. Loss Functions

1. **Dice Loss [41]**:
  Dice loss is based on the Dice coefficient and is commonly used for segmentation tasks. It measures the overlap between the predicted and true labels, focusing on improving performance for imbalanced datasets.
  (1) $$\mathrm{Dice}\;\mathrm{Loss}=1-\frac{2|X\cap Y|}{\left|X|+|Y\right|}$$
  where *X* and *Y* are the predicted and true sets, respectively.
2. **Focal Loss [41]**:
  Focal loss is designed to address class imbalance by down-weighting the loss assigned to well-classified examples, making the model focus more on hard-to-classify instances.
  (2) $$\mathrm{Focal}\;\mathrm{Loss}\left(p_{t}\right)=-\alpha {\left(1-p_{t}\right)}^{\gamma }\log\left(p_{t}\right)$$
  where $p_{t}$ is the predicted probability, α is a weighting factor, and γ is a focusing parameter.
3. **Weighted Dice Loss [42]**:
  Weighted Dice loss is a variation of Dice loss that assigns different weights to different classes, enhancing performance on datasets with imbalanced class distributions by penalizing certain classes more.
  (3) $$\mathrm{Weighted}\;\mathrm{Dice}\;\mathrm{Loss}=1-\frac{2\sum w_{i}x_{i}y_{i}}{\sum w_{i}x_{i}^{2}+\sum w_{i}y_{i}^{2}}$$
  where $w_{i}$ is the weight assigned to class *i*, and $x_{i}$, $y_{i}$ are the predicted and true values for class *i*.
4. **Combined Weighted Dice Loss [43]**:
  This is a hybrid loss that combines weighted Dice loss and CrossEntropy loss, allowing the model to balance overall performance while addressing class imbalances by tuning the contribution of each component.
  (4) CWDL=α·WDL+(1−α)·CrossEntropyLoss
  where CWDL is combined weighted Dice loss, WDL is weighted Dice loss, and α is a weighting factor to balance the two loss components.

It is found that the combination of combined weighted Dice loss and GeLU are the best performing. The combined weighted Dice loss performed the best across all activations (Table 1). However, it was found that the proposed study is able to squeeze more accuracy through the GeLU function.

### 4.3. Evaluation Metrics

For the evaluation part, the proposed study utilized the same metrics as in [16], namely Dice similarity coefficient (DSC) and accuracy, which frequently employed the performance of models. The DSC measures the overlap between predicted and actual results, particularly in segmentation tasks. Its mathematical formulation is given by:

(5) $$\mathrm{DSC}=\frac{2\cdot \mathrm{TP}}{2\cdot \mathrm{TP}+\mathrm{FP}+\mathrm{FN}}$$

The accuracy threshold determines whether the IoU score is adequate to consider a prediction correct.

(6) $$\mathrm{Accuracy}=\left\{\begin{array}{cc} 1 & \mathrm{if}\;\mathrm{IoU}\left(y_{\mathrm{true}},{\hat{y}}_{\mathrm{pred}}\right)>t_{\mathrm{IOU}}\\ 0 & \mathrm{otherwise} \end{array}\right.$$

The IoU metric is calculated using the following equation (Equation (7)).

(7) $$\mathrm{IoU}=\frac{\mathrm{TP}}{\mathrm{TP}+\mathrm{FP}+\mathrm{FN}}.$$

## 5. Results and Discussion

A key challenge in wave-based crack detection is differentiating between actual cracks and reflections caused by the sample’s border. The proposed model successfully learned to distinguish between these two cases, despite their similar wave behavior. When a wave encounters a crack, its interaction leads to a change in propagation, similar to when it reaches a boundary and reflects back. This distinction is particularly challenging because the returning wave from the border behaves similarly to one encountering a crack. Our model’s ability to differentiate these cases highlights its capability to extract nuanced temporal and spatial features. However, the model struggles when all crack sizes are included in the evaluation. This may be attributed to the scarcity of the smallest cracks in the dataset, leading to poor representation in the feature space. Additionally, smaller cracks may cause only subtle disruptions in wave behavior, making them harder to detect.

### 5.1. MDA Analysis

Manifold discovery and analysis (MDA) helps visualize the higher dimensional manifolds formed by the intermediate layers of the model in lower dimension [15]. These plots help visualise the learned features in the *ℓ*th layer with respect to the output manifold. Unlike methods such as t-SNE and UMAP, which only work on classification tasks, MDA works on regression tasks, where the output manifold can have a complex shape. MDA also preserves the geodesic distances between higher dimensional feature points, preserving both local and global structure.

In a nutshell, MDA works as follows: First, distance is computed between the estimated outputs of the DNN, from this distance, the farthest point is chosen to construct the boundary of the output manifold. All the points are sorted w.r.t the farthest point in k bins using the optimal histogram bin count. These bins become the labels that will be used in the second step. Second, the high-dimensional features from an intermediate layer are projected to the manifold using the Bayesian manifold projection (BMP) approach. BMP computes a posterior distribution over the low-dimensional space by combining the prior (based on pseudo-labels and manifold structure) with a likelihood (based on the observed data). Finally, a DNN trained on predicting the location of uncertain Bayesian points on a 2D embedding space is used to visualise the results. The plots are assessed qualitatively on the following points:

- Feature Separation and Continuity: The MDA visualization shows a curved shape, indicating that the features extracted from the neural network follow a smooth continuum along the manifold. This suggests that the neural network is capturing meaningful information.
- Color Gradient: A spectrum of gradients is shown, implying that the model has learned to separate different features.

**Figure 13:**
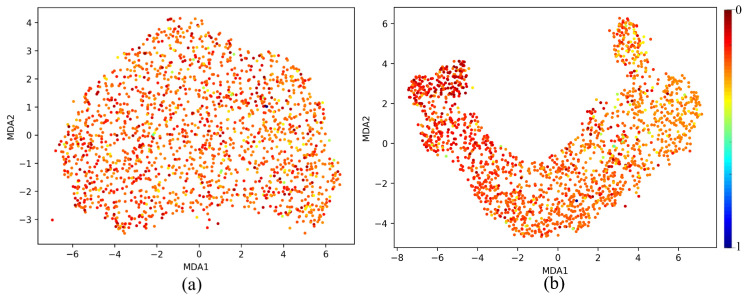
MDA visualization of Layer 64 comparing (**a**) untrained model and (**b**) trained model.

**Figure 14:**
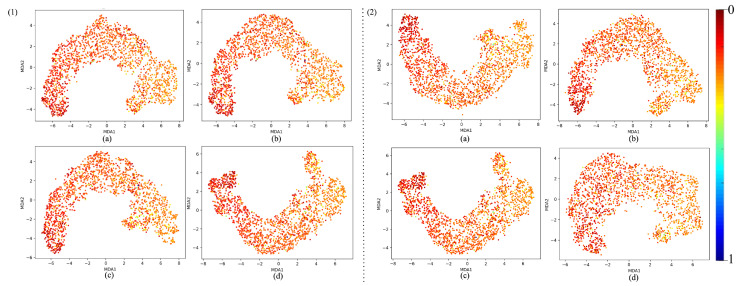
(**1**) MDA visualizations of layers using Gelu activation and Dice loss, shown for (**a**) Layer 22, (**b**) Layer 25, (**c**) Layer 34, and (**d**) Layer 64 and, (**2**) MDA visualization of Layer 64 utilizing different activation functions: (**a**) ELU, (**b**) ReLU, (**c**) GELU, and (**d**) SELU.

**Figure 15:**
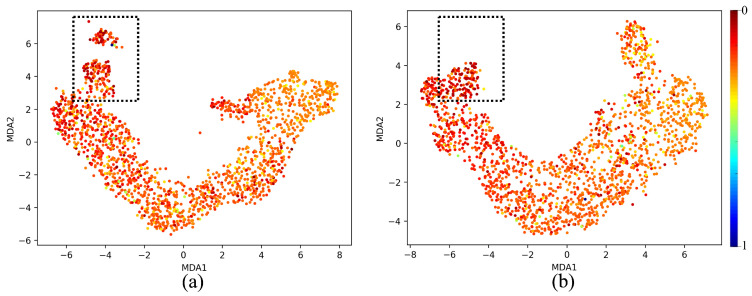
MDA visualization comparing (**a**) 1D Densenet [16] and (**b**) proposed model—MicrocrackAttentionNext. The highlighted region in black indicates the region where the cluster is broken in 1D Densenet. In contrast, the same region in MicrocrackAttentionNext shows coherency, implying that the MicrocrackAttentionNext learned good feature representations for microcracks.

The MDA plot Figure 13a for the untrained model shows a relatively disorganized and diffuse clustering of points. This suggests that the feature representations at Layer 64 are not yet structured in a meaningful way to distinguish between patterns within the dataset. The absence of clear separation or distinct clustering patterns indicates that the untrained model has not yet learned to capture the underlying structure of the data, which is expected at the initial stages of training. At this stage, the network’s representations are largely random, as it has not yet learned the task-specific features. The spread-out nature of the points highlights that the model is treating all inputs similarly, without any differentiation based on the features it should detect. In contrast, the MDA plot Figure 13b for the trained model reveals a much more structured and organized distribution. The *ℓ*th layer of a well-trained model shows the cluster with a smooth arch-like structure and a gradient of colors differentiating the two output extremums; red representing 0 and blue representing 1. The analysis of various layer depths and activation functions in the MicrocrackAttentionNext reveals a clear pattern in the network’s ability to form distinguishable manifold curves. Figure 14(1) visualizes the manifold at different layers of the network. All the layers show consistent and smooth arch-like shapes. This implies that all the layers have learned good representations. In Figure 14(2), effects of various activations are plotted, and it is found that ELU shows a more spread out cluster especially toward the light colors (towards crack class) and ReLU shows a good arch-like structure with tightly packed dots of red color (no crack class). Still, the light color dots are much more incoherent and less compact. SeLU shows a poorly defined structure compared to all other activations. This is also reflected in the results table, where SeLU performs worse in all cases. This behavior of SeLU can be attributed to its self-normalizing property, as it forces all outputs to behave similarly, “dampening” the importance of smaller, rare patterns. This makes the model less discriminative, making the plot less defined and incoherent. In terms of cluster shape and compactness, GeLU is similar to ReLU, which should be the case, as GeLU is the smoothed version of ReLU. Very similar performance in the results table is also observed. Among all the activations, GeLU performed the best; this fact also reflects on the MDA plot where the cluster is very smooth and the lighter points are more compactly packed relative to other activation functions. Its smooth probabilistic gating mechanism helps in finely controlling how information is passed through the network, allowing the model to focus more on the minority class. Figure 15 shows MDA visualization of the 1D Densenet proposed in [16] compared with the proposed MicrocrackAttentionNext model. One thing to note in all of the above MDA plots is the absence of a full spectrum of colors in the plot. This is mainly attributed to the severe class imbalance in the data. This class imbalance leads to very few values in the feature map strongly correlating to the strong value of predicting the crack class. This is further aggravated by the dimensionality reduction, which renders even fewer points corresponding to higher confidence values. Hence, only one blue point is seen. The proposed MicrocrackAttentionNext achieved a DSC of 0.91. Table 2 shows the comparison of different loss functions used to train MicrocrackAttentionNext.

### 5.2. Thresholding Analysis in Prediction Accuracy

Pixel-level predictions were binarized using a threshold of 0.5, where values exceeding this threshold indicated the presence of a crack. Prediction accuracy was assessed based on the intersection over union (IoU) metric, with a threshold of 0.5 selected to determine accurate crack identification. This threshold ensures a balance between minimizing false positives and maintaining practical accuracy.

**Figure 16:**
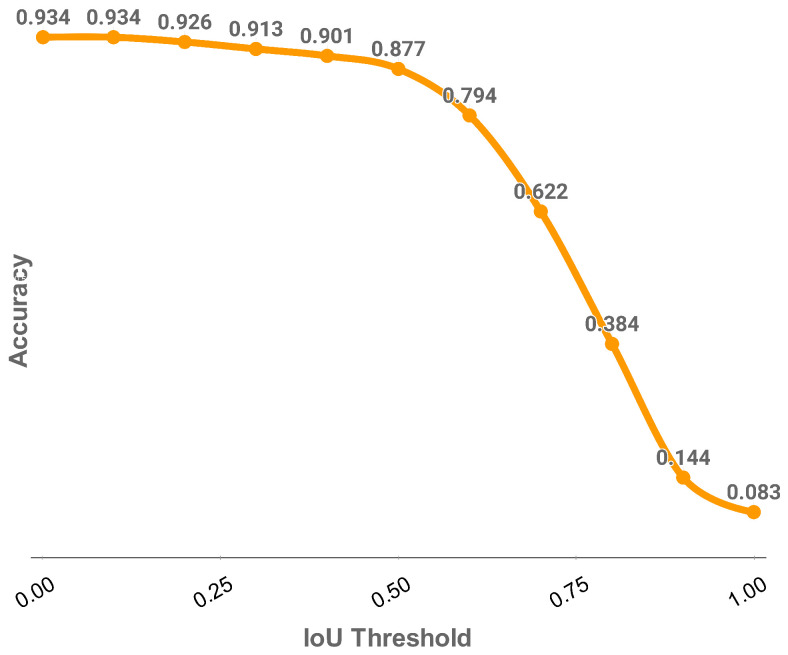
Effect of IOU threshold on accuracy for bin threshold at 0.5 and inclusive of all crack size.

Figure 16 demonstrates the relationship between varying IoU thresholds and overall accuracy. An IoU value approaching 1 requires near-perfect pixel classification but results in a significant accuracy drop due to its stringent nature. Conversely, an IoU value near 0 allows for minimal correct pixel identification but fails to filter out false positives. The chosen IoU threshold of 0.5 provides a practical compromise, achieving reasonable accuracy while maintaining meaningful crack identification.

**Figure 17:**
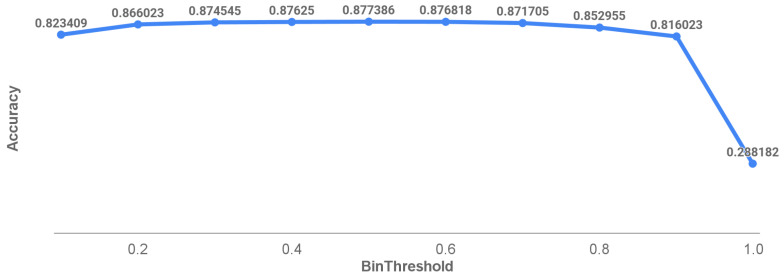
Binarising threshold when IoU threshold is 0.5.

Figure 17 explores the influence of both IoU and binarizing thresholds on accuracy, holding one threshold constant at 0.5. Variations in the binarizing threshold showed minimal impact on accuracy, except at extreme values (near 0 or 1), indicating strong confidence in the model’s pixel-level predictions.

### 5.3. Comparison with Similar Works

The uniqueness of this work lies in the dataset used and the applied deep learning algorithms. The data, which were exclusively collected at the researcher’s university, consist of purely numerical values, distinguishing them from the image data commonly used in the literature. To the best of our knowledge, no deep learning models have been trained on this type of numerical data. The goal is to develop models that not only detect cracks but also precisely segment them. Additionally, the presence of multiple crack sizes increases complexity, as the behavior of the wave differs depending on the crack dimensions.

The earlier implemented models can serve as benchmarks for comparison in this work. These previous models were applied to the same dataset and provide a foundation for evaluating the performance of the proposed deep learning approaches. The results from these models allow us to objectively assess the progress and improvements made with the new algorithms.

Table 1 presents a comparison of the performance of the earlier models [16,26,27] with the current results.

The proposed method, **MicrocrackAttentionNext**, achieves an accuracy of **0.8777**, an IoU of **0.8521**, and a DSC of **0.9145**. These results outperform previous models on key metrics, suggesting that our approach is not only highly accurate but also effective at localizing cracks. A higher IoU indicates that the predicted crack regions overlap closely with the ground truth, while the DSC improvement reflects enhanced segmentation performance. However, the model’s precision (**0.8601**) and recall (**0.8518**) indicate room for improvement. In particular, there remains scope to reduce false positives and false negatives. Future work will involve further tuning of the network architecture, incorporating additional data, and exploring advanced loss functions to address these shortcomings.

**Figure 18:**
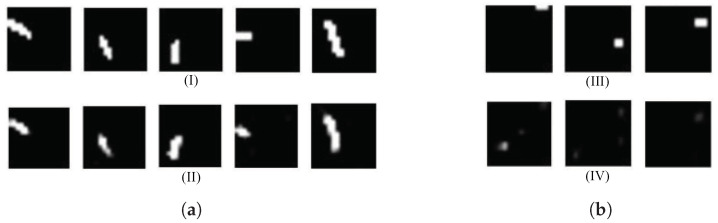
(**a**) Positive examples from MicrocrackAttentionNext (I) represents the ground truth and (II) the corresponding model prediction. (**b**) Failure sample (III) represents the ground truth, while (IV) shows the corresponding model prediction.

The proposed model provides better performance on DSC and IoU metrics, suggesting that the proposed model is not only able to detect the crack with high accuracy but also localize it better. A higher IoU score also suggests that the predicted crack region and the ground truth region have a high overlap, meaning the model is drawing the crack boundary closer to where the crack actually exists. With better DSC and IoU the model is also less likely to make mistakes and detect cracks where there are no cracks present (false positives) or miss cracks that should be detected (false negatives). This is important for real-world applications, such as structural health monitoring where accuracy is critical. Figure 18a shows successful segmentation from MicrocrackAttentionNext while Figure 18b shows some failure segmentation samples.

Table 3 compares the models on the basis of training time, number of layers, and parameters. This table illustrates the computational complexity and efficiency of each architecture. For instance, while some models (such as 1D-DenseNet-TConv originally trained for 200 epochs) have a significantly larger number of layers and require more training time, our proposed model maintains competitive performance with considerably fewer layers and reduced training time. Such efficiency is critical for deployment in environments with limited computational resources.

Furthermore, Table 4 provides a breakdown of the segmentation accuracy with respect to various crack sizes (measured in micro meters). This detailed analysis shows that the proposed model maintains high accuracy across all crack size ranges. For instance, while the accuracy in the 0–3 µm range is moderate (0.5214), it improves significantly for larger cracks, reaching up to 0.9917 in the 9–14 µm range. This indicates that the model is particularly effective at localizing larger cracks, although there remains potential to improve performance on very fine cracks. Addressing this could be a focus for future model refinement, possibly through specialized training strategies or additional data augmentation techniques.

## 6. Conclusions and Future Work

### 6.1. Conclusions

In this study, we introduced MicrocrackAttentionNext, an advanced deep learning model designed for microcrack detection in wave field analysis. The key novelty of this model lies in its Adaptive Feature Reuse Block, which significantly improves feature utilization across different crack sizes. This block helps the model focus on the most informative features, enhancing detection accuracy while reducing the risk of overfitting, particularly when dealing with small and subtle cracks. Through a series of experiments, we compared various model configurations, including baseline models and models with different architectural adjustments. The experimental results indicated that MicrocrackAttentionNext outperforms previous models in multiple metrics, with notable improvements in the Dice similarity coefficient (DSC) and intersection over union (IoU), which directly correlate with better crack localization and segmentation. Specifically, the model achieved an accuracy of 87.77% overall, surpassing the previous benchmarks, and demonstrated a DSC of 0.9145 and IoU of 0.8521, suggesting a superior ability to precisely detect and delineate cracks.

However, despite these significant advancements, the precision and recall values of the proposed model (0.8601 and 0.8518, respectively) indicate room for further refinement, particularly in reducing false positives and false negatives. To this end, future work will focus on fine-tuning the network architecture and exploring the integration of additional data sources to address these gaps. One possible avenue for improvement is the introduction of more advanced loss functions tailored to handle class imbalance more effectively, especially for detecting small cracks that introduce minor perturbations in the wave field. Moreover, extending the model’s training with more epochs and experimenting with alternative attention mechanisms could potentially yield further improvements in both the overall performance and handling of smaller crack sizes.

The model is capable of segmenting the microcracks, allowing us to determine their spatial locations in the material. The qualitative examination of the activation functions using the manifold discovery and analysis (MDA) algorithm allowed the evaluation of the impact of different activation and loss functions on the model’s performance. The proposed model and 1D-Densenet were analyzed using the MDA plots. The manifold of the proposed model was more compact with a much smoother arc than the 1D-Densenet.

### 6.2. Future Work

In future efforts to improve microcrack detection models, two primary strategies can be pursued: expanding datasets and refining model architectures. The dataset used presents a challenge due to severe class imbalance, which requires more advanced techniques for data generation and augmentation to mitigate the bias introduced. Moreover, the segmentation output is limited by low resolution and without appropriate upscaling techniques critical details may be lost. Although the encoder architecture performs well enough, more changes are necessary in the decoder section of the segmentation model to achieve improved results and maintain consistency with high-quality input features. Expanding the dataset to include a wider range of crack patterns and sizes will further enhance the robustness of the model. Larger and more diverse datasets with multiple cracks in a single sample will help address class imbalance, improve generalization, and refine the model’s ability to distinguish subtle crack patterns. Additionally, a more balanced dataset containing a greater number of small cracks would help the model better capture small wave disturbances. Since smaller cracks may introduce only minor perturbations in wave behavior, their detection is more challenging. Data from multiple excitation points for a single sample will enhance the quality of the dataset. To enhance the encoder’s ability to capture long-range dependencies, a state space model can be used, integrating the recently proposed Mamba architecture in particular. This adjustment could improve the model’s ability to handle complex spatial relationships, thereby strengthening feature extraction and contributing to overall performance gains in the segmentation task.

## Figures and Tables

**Table 1:** Comparison against previous works on the same dataset.

Literature	Accuracy	IoU	DSC	Precision	Recall
1D-DenseNet-TConv (200 epochs)	0.8368	0.7601	0.8637	0.8753	0.8523
MicroCracksAttNet (50 epochs)	0.8601	0.7666	0.8684	0.8811	0.8888
MicroCracksMetaNet (50 epochs)	0.867	0.8082	0.8943	0.9066	0.8911
**Proposed** **MicrocrackAttentionNect** **(50 epochs)**	**0.8777**	**0.8521**	**0.9145**	**0.8601**	**0.8518**

**Table 2:** Comparison of accuracies using different loss functions for multiple crack sizes. FL: focal loss, DL: Dice loss, WDL: weighted Dice loss, CWDL: combined weighted Dice loss.

Activation Function	Loss Function	>0 μm	>1 μm	>2 μm	>3 μm	>4 μm
GeLU	FL	0.8275	0.8612	0.9354	0.9501	0.9541
	DL	0.8633	0.9012	0.9585	0.9701	0.9802
	WDL	0.8381	0.8798	0.9415	0.9670	0.9793
	CWDL	**0.8774**	**0.9211**	**0.9814**	**0.9808**	**0.9848**
ReLU	FL	0.8252	0.8632	0.9456	0.9701	0.9802
	DL	0.8553	0.8902	0.9646	0.9770	0.9829
	WDL	0.8213	0.8687	0.9293	0.9524	0.9703
	CWDL	0.8678	0.9134	0.9673	0.9808	0.9866
ELU	FL	0.8313	0.8797	0.9558	0.9839	0.9911
	DL	0.8502	0.9011	0.9673	0.9831	0.9884
	WDL	0.8563	0.9034	0.9605	0.9739	0.9829
	CWDL	0.8515	0.9041	0.9673	0.9847	**0.9920**
SeLU	FL	0.8206	0.8671	0.9503	0.9793	0.9902
	DL	0.8412	0.8993	0.9707	**0.9870**	0.9893
	WDL	0.8201	0.8664	0.9307	0.9555	0.9712
	CWDL	0.8443	0.8910	0.9625	0.9854	0.9929

Best result for a crack size is highlighted with bold.

**Table 3:** Training performance and architectural complexity comparison among four deep learning models: MicroCracksAttNet, MicroCracksMetaNet, 1D-DenseNet-TConv, and MicrocrackAttentionNext.

Attributes	MicroCracksAttNet	MicroCracksMetaNet	1D-DenseNet-TConv	MicrocrackAttentionNext
**Layers**	58	72	444	94
**Epochs**	50	50	200	50
**Total params**	1,129,209	1,131,690	1,393,429	1,280,299
**Trainable params**	1,127,321	1,129,722	1,376,137	1,277,355
**Non-trainable** **params**	1888	1968	17,292	2944
**Time taken by** **first Epoch**	26.45 s	42.35 s	89.14 s	36.98 s
**Total training** **time**	906.33 s	2117.50 s	15,560.56 s	1849.54 s

**Table 4:** Crack detection accuracy measured across crack size intervals (in micrometers).

Model	Crack Sizes μm			
	**0–3 μm**	**3–6 μm**	**6–9 μm**	**9–14 μm**
MicrocrackAttentionNext (50 epochs)	0.5214	0.9787	0.9879	0.9917
MicroCracksMetaNet (50 epochs)	0.4513	0.9549	0.9753	0.9862
MicroCracksAttNet (50 epochs)	0.4420	0.9587	0.9778	0.9862
1D-DenseNet-TConv (50 epochs)	0.3719	0.9268	0.9778	0.9835
1D-DenseNet-TConv (200 epochs)	0.4682	0.9793	0.9852	0.9972

## Data Availability

The data supporting the findings of this study are not publicly available due to privacy restrictions. Access to the data can be considered upon reasonable request to the corresponding author, subject to institutional and ethical guidelines.

## Abbreviations

The following abbreviations are used in this manuscript:

AE	Acoustic Emission
CNN	Convolutional Neural Network
CWDL	Combined Weighted Dice Loss
DL	Dice Loss
DSC	Dice Similarity Coefficient
DNN	Deep Neural Network
FL	Focal Loss
GELU	Gaussian Error Linear Unit
IoU	Intersection over Union
LEM	Lattice Element Method
MDA	Manifold Discovery and Analysis
MDPI	Multidisciplinary Digital Publishing Institute
NDT	Non-Destructive Testing
ReLU	Rectified Linear Unit
SAW	Surface Acoustic Wave
SE	Squeeze-and-Excitation
SELU	Scaled Exponential Linear Unit
TConv	Transposed Convolution
TOFD	Time-of-Flight Diffraction
WDL	Weighted Dice Loss
